# Role and Perspectives of Inflammation and C-Reactive Protein (CRP) in Psychosis: An Economic and Widespread Tool for Assessing the Disease

**DOI:** 10.3390/ijms222313032

**Published:** 2021-12-02

**Authors:** Irfan Ullah, Hashir Ali Awan, Alifiya Aamir, Mufaddal Najmuddin Diwan, Renato de Filippis, Sana Awan, Muhammad Irfan, Michele Fornaro, Antonio Ventriglio, Federica Vellante, Mauro Pettorruso, Giovanni Martinotti, Massimo Di Giannantonio, Domenico De Berardis

**Affiliations:** 1 Department of Internal Medicine, Kabir Medical College, Gandhara University, Peshawar 25000, Pakistan; irfanullahecp2@gmail.com; 2 Department of Internal Medicine, Dow Medical College, Karachi 74200, Pakistan; hashiraliawan@gmail.com (H.A.A.); alifiya.aamir521@gmail.com (A.A.); mufdiwan@gmail.com (M.N.D.); sana.awan527@gmail.com (S.A.); 3 Department of Health Sciences, University Magna Graecia of Catanzaro, Viale Europa, 88100 Catanzaro, Italy; defilippisrenato@gmail.com; 4 Department of Internal Medicine, Hayatabad Medical Complex, Peshawar 25000, Pakistan; irfanjamal2k18@gmail.com; 5 Department of Psychiatry, Federico II University, 80131 Naples, Italy; dottfornaro@gmail.com; 6 Department of Experimental and Clinical Medicine, University of Foggia, 71122 Foggia, Italy; a.ventriglio@libero.it; 7 Department of Neurosciences and Imaging, Chair of Psychiatry, University “G. D’Annunzio”, 66100 Chieti, Italy; federica.vellante@gmail.com (F.V.); mauro.pettorruso@hotmail.it (M.P.); giovanni.martinotti@gmail.com (G.M.); digiannantonio@unich.it (M.D.G.); 8 Department of Mental Health, Psychiatric Service for Diagnosis and Treatment, Hospital “G. Mazzini”, ASL 4, 64100 Teramo, Italy

**Keywords:** c-reactive protein (CRP), inflammation, psychosis, schizophrenia

## Abstract

Schizophrenia is a major psychotic disorder affecting nearly 23.6 million people globally and greatly impacting the cognitive and social functioning of individuals. Multiple risk factors, including genetic, environmental, and epigenetic factors have been identified. However, the exact mechanism by which some factors aid in the development of schizophrenia is still uncertain. Acute and/or long-standing inflammation has been implicated as both a cause and effect of schizophrenia. Heightened immune responses have been documented in large cohorts of individuals with schizophrenia. While not completely known, multiple hypotheses, such as disruption of the blood–brain barrier, alterations in the kynurenine/tryptophan pathway, and increased microglial activation, have been presented to correlate inflammation with schizophrenic symptoms. Measurement of C-reactive protein (CRP) is a commonly performed and inexpensive test on patients’ serum to determine levels of systemic inflammation in the body. Multiple studies have reported an elevated CRP level in different stages of schizophrenia, indicating its potential to be used as a viable biomarker in the diagnosis and monitoring of schizophrenia along with assessing treatment response to conventional and non-conventional treatment regimens. This review aims to evaluate the role of inflammation, in general, and CRP, in particular, in the pathogenesis of schizophrenia and its potential significance in diagnostic, therapeutic, and preventative approaches towards schizophrenia and psychosis.

## 1. Introduction

Psychosis is a psychiatric disease condition that results in not being able to distinguish internal stimuli from external ones. This occurs with some degree of confusion, dysfunction, and disorientation with reality [1]. This may occur as a consequence of various psychiatric, medical, or neurological conditions [1]. While most psychoses have a psychiatric origin, they may also be drug-induced or manifest due to co-occurring medical or neurological illness [2]. Mood disorders like major depressive disorder (MDD) or bipolar disorder may also have psychotic components or features [3]. However, despite the seemingly wide range of psychiatric and non-psychiatric situations that may result in this state, the most important subset of disorders of which psychosis is a fundamental and central feature are psychotic disorders [1]. Sometimes referred to as “primary” psychotic illnesses, the hallmark symptom of these disorders is a substantial degree of psychosis unexplained by any other reason or underlying condition [3,4]. Among these psychotic disorders, the most prominent and prevalent is schizophrenia.

The Global Burden of Diseases study in 2019 [5] recorded the incidence of schizophrenia to be 16.3 cases per 100,000 people and reported a total of 23.6 million cases worldwide. Furthermore, schizophrenia constituted 12.2% of all disability-adjusted life years (DALYs) caused by mental health disorders [5]. Even though schizophrenia is considered to have a low prevalence [6,7], within the last decade, a 14% increase was recorded in the global prevalence of schizophrenia [5]. While some of the highest incidence rates of schizophrenia were found in high-income countries (demarcated according to a sociodemographic index) [5], the massive economic burden carried by this condition has the potential to substantially impact low- and middle-income countries (LMICs) [7].

In the latest edition of the Diagnostic and Statistical Manual of Mental Disorders (DSM-5), the spectrum of psychotic disorders like schizophrenia, schizophreniform disorder, and brief psychotic disorder are delineated by the duration of psychotic symptoms in each [2]. Schizophrenia has a symptomatic presentation in line with that of psychosis and its elements lasting for six months or more [2]. Symptomatic elements of psychosis are listed in the DSM-5 to assist in the identification and assessment of psychotic disorders. These include delusions, hallucinations, disorganized thinking, disordered motor response, and negative symptoms [2]. Symptoms are also generally separated into two areas: positive (such as delusions and hallucinations) and negative (such as inability to feel pleasure, known as anhedonia, and poverty of speech, known as alogia) [6].

There are multiple risk factors that predispose individuals to develop a psychotic disorder like schizophrenia. Genetic contributions are elucidated by a 46% chance of both monozygotic twins (having the identical genetic makeup) developing schizophrenia and a 40% increased risk of children developing schizophrenia if both parents are affected [6]. On the other hand, unique environmental insults and other developmental risk factors are reported to influence an individual’s chances of developing psychotic symptoms. Maternal factors like complications during delivery, birth season, malnutrition, and infections, along with personal factors like residing in urbanized locations, having a history of substance abuse or addiction, experiencing childhood trauma, and belonging to a minority ethnic community are known to play a crucial role and affect the vulnerability of an individual [6,8]. However, genetic tendencies and environmental factors are not considered as completely discrete. Instead, an intricate interplay among them is suspected beyond a simplistic “two-hit” hypothesis which is generally proposed [8] that also involves epigenetics [9]. As a result of widely varying factors and their complex relationship with each other, the causality of schizophrenia exhibits considerable heterogeneity and inconsistency [8].

## 2. Systemic Inflammation as a Risk Factor for Schizophrenia

Evidence from population-based cohort studies has linked a history of systemic inflammation in gestational, neonatal, or early childhood stages of an individual with increased vulnerability to developing psychoses. A ramped-up immune response in the mother during pregnancy in response to infections will invariably lead to a greater in utero inflammatory exposure to the fetus [10]. A systematic review on ascertaining the effect of prenatal infections on the risk of developing schizophrenia in adult life revealed that an individual becomes two to five times more vulnerable to schizophrenia if there is a history of neonatal infection of multiple microbes [11]. The review also commented that prenatal exposure to inflammatory molecules, especially tumor necrosis factor (TNF), also contributes to schizophrenia risk [11,12]. High levels of inflammation in mothers during pregnancy have been associated with an increased risk of schizophrenia in the child even after adjusting for known risk factors like urbanization and socioeconomic status [13]. Apart from prenatal infections, a meta-analysis revealed that early childhood illnesses, especially viral central nervous system (CNS) infections, were found to double the risk of said subjects developing schizophrenia [14]. On the other hand, non-infective causes of extensive inflammation like autoimmune diseases are also known to impact the development of psychosis in individuals [15,16].

## 3. Pathophysiology of Schizophrenia and the Role of Inflammation

On a molecular level, psychotic disorders like schizophrenia are postulated to arise due to a disbalance and abnormality in neurotransmitter levels. Dopamine and dopaminergic pathways are primarily implicated, but glutamate, gamma-amino-butyric acid (GABA), and acetylcholine are also presumed to play a vital role [1,17]. Other hypothesized mechanisms for the development of schizophrenia include changes in neuroanatomy and abnormalities in neurological development [6]. Even when the concept of inflammation causing psychosis was coined in the late 19th century, promising advances in the field of immunopsychiatry have led to mounting evidence regarding the part played by local and systemic inflammation in the development and continuation of neuropsychiatric illnesses including psychotic disorders like schizophrenia [18].

The brain was historically considered to possess “immune privilege” via its blood–brain barrier (BBB) that spared it from potentially detrimental effects of widespread systemic inflammation. However, recent findings have suggested that the brain may not be entirely protected from the non-specific innate inflammatory response [19], and “complex brain-immune interactions” take place by the action of circulating inflammatory molecules [18]. Evidence suggests that the brain is affected by a heightened immune response or extensive inflammation due to infections (especially of neurotropic viruses like SARS-CoV and SARS-CoV-2) or pro-inflammatory conditions [20,21,22]. This may lead to a local microglial response, increased cytokines and chemokines, and encephalitis which alters cognitive ability, behavioral response, and result in neurological deficits [18,23].

Research on the pathophysiology of MDD revealed a high amount of circulating immune mediators, including cytokines, that interacted with, and invariably impacted, neurotransmitter levels and neuroplasticity [24]. It is assumed that common pathways involving immune response and a genetic predisposition allow enhanced inflammation to affect neurodevelopment and brain function in the development of schizophrenia as well [10,18,25]. Genes, including the major histocompatibility complex (MHC) region on chromosome 6, involved in the immune and inflammatory response are known to be mutated via copy-number variations or single-nucleotide-polymorphisms in schizophrenia [26].

The role of autoantibodies against the N-methyl-D-aspartate (NMDA) receptor is also linked with the appearance of psychiatric illnesses including schizophrenia [25,27,28]. Being a receptor for the neurotransmitter glutamate, the hypofunction of the NMDA receptor due to the action of autoantibodies decreases the glutamate transmission through synapses. Interestingly, blocking of NMDA receptors via ketamine-induced psychotic symptoms resembling schizophrenia [29,30]. Kynurenic acid is an endogenous antagonist of NDMA receptors that is produced via the kynurenine pathway involved in tryptophan degeneration [31,32]. Trytophan metabolized via the indoleamine 2,3-dioxygenase (IDO) pathway or the tryptophan 2,3-dioxygenase (TDO) pathway, although at low levels, leads to the production of kynurenic acid in the CNS itself utilizing enzymes present inside astrocytes [33]. Apart from NDMA, kynurenic acid also inhibits kainate and AMPA receptors. On NDMA, kynurenic acid attaches with the greatest affinity to the glycine co-agonist site [34].

Systemic inflammation ramps up the production of tryptophan metabolites and precursors of kynurenic acid (such as kynurenine) peripherally. These can easily pass through the BBB and enter the CNS via the circulation [33]. Kynurenine is then converted to kynurenic acid in astrocytes [33]. It is important to note that kynurenine can pass through the BBB while kynurenic acid cannot and therefore it is formed inside the CNS only [34]. Furthermore, in settings of increased neuronal cytokine activity, the production of kynurenic acid is highly stimulated, showing how it is largely “inflammation-driven” [31,34] and studies have found an elevated kynurenic acid level in patients of schizophrenia, especially within CNS [35]. Antagonistic activity of kynurenic acid on the NMDA receptor can be one of the reasons why increased cytokine activity in the brain may lead to schizophrenic symptoms [18]. As described earlier in this text, neurotransmitter imbalance has been implicated in the causality of schizophrenia, and NMDA receptor hypofunction is hypothesized to be responsible for hyperdopaminergia due to upstream disinhibition of excitatory neurons [34,36]. A study on twins also concluded that kynurenic acid and other inflammatory metabolites were correlated in psychosis as elucidated by significantly higher levels in the affected twin [37].

On the other hand, mouse models have provided insight into the nature of systemic inflammation’s effects on the brain and the mechanisms by which it alters functions. Some have implicated retrograde neuronal transport and subsequent activation of local microglial response with release of cytokines and other mediators, while others have brought into focus the possibility of microglial priming after early infections leading to a greater response in case of any future systemic inflammation [18]. Increased oxidative stress via microglial activation may accelerate neurodegeneration as well as cause a cognitive decline classically seen in long-standing schizophrenia [18].

## 4. Role of CRP in Development of Schizophrenia

C-reactive protein (CRP) is an acute-phase protein that is produced by hepatocytes and other cell types, including immune cells, endothelial cells, and smooth muscle cells, after stimulation of the gene encoding for CRP by Interleukin-6 (IL-6) [38]. The circulating levels of CRP in serum rise and fall according to the inflammatory status of the body and therefore it is the most commonly used biomarker of systemic inflammation worldwide [38,39]. CRP is a standard laboratory exam and can be measured in the peripheral blood and analyzed in any clinical laboratory around the globe. Therefore, CRP has a very promising potential in being a clinical biomarker for psychiatric disorders [40]. The high-sensitivity CRP (hs-CRP) assay has a lower limit of detection of 0.1 mg/L. However, these detection limits may vary from manufacturer to manufacturer. The measurement of CRP is useful in the diagnosis and monitoring of many acute and chronic inflammatory conditions of both infectious and non-infectious etiology [41].

The role of CRP in the causality of schizophrenia has been a subject of interest for many years. It is known that CRP and other acute-phase reactants cause disruption of the BBB and alter its permeability for inflammatory mediators and antibodies [42,43]. As described above, this compromise in the integrity of the BBB in the backdrop of widespread systemic inflammation has been linked to developing psychotic symptoms [15,25]. Increased CRP has also been recently linked with a significant decline in multiple cognitive domains, including working memory and learning ability, in individuals of all ages suffering from schizophrenia [44]. Further reviews were consistent in highlighting a marked cognitive impairment in schizophrenic patients with even low-grade or subclinical inflammation [45,46]. Interestingly, a study in Finland elucidated the role of elevated maternal CRP levels in increasing the risk of psychosis in the offspring [13]. In summary, an inverse association of CRP levels and cognitive ability is noted in acute psychosis. However, a clinical trial published in 2019 provided valuable insight into the prognostic value of CRP in six months following the resolution of acute psychosis [47]. Cognitive abilities improved with time and a noticeable drop in CRP levels was noted in the later periods, continuing the inverse relationship [47]. The pathophysiology behind increased CRP levels and subsequent cognitive dysfunction is not fully known but some studies have implicated CRP in increasing BBB permeability during acute inflammatory phases and causing neuroinflammation [48]. Mouse models revealed that CRP has no virtual effect on BBB permeability in smaller amounts but impairs its function and increases paracellular permeability in higher amounts that may be achieved during high systemic inflammation or pro-inflammatory states like psychosis [48]. Multiple complex molecular pathways have been described to explain CRP-led increased permeability of the BBB and endothelial dysfunction [48]. Notably, it is widely believed that the disruption of tight junctions is involved in the increased permeation of CRP [43]. CRP in blood activates the surface Fc-gamma receptors (CD16/32) on endothelial cells and leads to the formation of reaction oxygen species (via p38-mitogen-activated protein kinase mechanism) that causes an alteration in the myosin light chain kinase activity [43]. This implies that impairment of tight junctions by CRP is most likely achieved via modification of the cytoskeletal structure and induction of abnormal contractility [43,48]. After entering the CNS, CRP causes a reactive microglial reaction, astrogliosis, and neuroinflammation [49]. Neuroinflammation in psychiatric conditions is invariably associated with short-term and lasting cognitive deficits [50]. Therefore, this mechanism may explain how elevated CRP levels (reflecting systemic inflammation) in psychosis may lead to decreased cognitive abilities.

**Figure 1:**
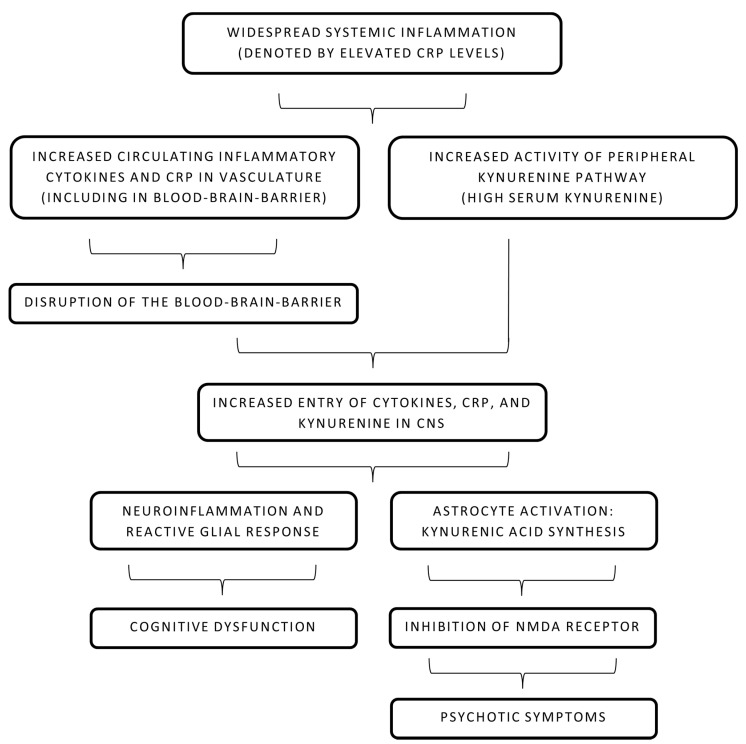
Pathophysiology of systemic inflammation and schizophrenia. CNS: central nervous system, CRP: C-reactive protein, NMDA: N-methyl-D-aspartate.

Apart from CRP’s direct role in the disruption of the BBB, its role in the increment of the kynurenine pathway is still unclear. High kynurenic acid levels, as described earlier in this text, act as an antagonist at the NMDA receptor and possibly contribute to psychotic symptoms [28]. Both CNS and peripheral kynurenine pathways are tightly regulated by the immune status of the body [33]. With increased BBB permeability in the setting of elevated CRP levels, infiltration of CRP and other cytokines in CNS leads to neuroinflammation and increased activation of astrocytes and microglia [51]. Tryptophan degeneration and kynurenic acid production are increased and the antagonistic effect on NMDA is more pronounced, leading to psychotic symptoms [33]. It is also important to consider that some studies have concluded that CRP levels are not associated with the kynurenine pathway or levels of kynurenic acid in CNS [52,53]. It is important to note that CRP levels have been largely associated with cognitive symptoms in psychosis, while kynurenic acid levels are associated with psychiatric symptoms [54]. Therefore, it is unclear whether CRP levels and kynurenic acid levels act as two independent markers occurring coincidentally during schizophrenia or have a complex interaction that leads to a possible cause–effect relationship. Figure 1 attempts to cohesively summarize both pathways discussed in this section: greater CRP levels causing cognitive dysfunction and a heightened immune response leading to activation of the kynurenine pathway. The subsequent astrocyte-dependent conversion to kynurenic acid is also depicted.

## 5. Associations of CRP with Psychotic Symptoms and Role in Clinical Evaluation

A review of current findings necessitates further research on a molecular level that illustrates the processes behind the appearance of psychotic symptoms in patients with high CRP levels. However, Mendelian randomization (MR) studies on CRP and risk of developing schizophrenia have mostly shown CRP to have a protective causal effect. Ligthart believes it is due to the conventionally accepted antimicrobial qualities of CRP that allow avoidance of childhood infections, which is one of the risk factors of developing psychosis [55].

Apart from cognitive deficits, CRP levels have been associated with a variety of psychotic symptoms and different forms of schizophrenia. An association of CRP levels and the appearance of negative symptoms was also found via a cross-sectional study [56]. Additionally, a recent systematic review recorded elevated CRP levels to be notably associated with positive symptoms of acute psychosis seen in schizophrenia [39]. Furthermore, the findings showed an increased serum level of CRP may also be used to predict the onset of schizophrenia along with various cognitive and physical complications of schizophrenia [39]. Inability to distinguish meaningful sensory stimuli from others, known as sensory gating deficit, has also been associated with elevated CRP levels in psychosis [57]. However, it is important to note that findings of individual cross-sectional studies conducted to investigate the association of CRP and psychotic symptoms manifesting in schizophrenia show considerable heterogeneity and variable conclusions [39]. Moreover, the findings of this systematic review by Fond et al. are inconclusive about the exact role of CRP [39]. While an increased CRP level is noted, it is unclear whether this is an effect of schizophrenia or it is directly involved in the pathogenic mechanism behind it [39].

However, it is crucial to note that the findings of the systemic review are in line with conclusions of an earlier meta-analysis consisting of 1963 patients with schizophrenia compared with 3683 non-schizophrenics [58]. A modest rise in CRP levels was associated with schizophrenia in comparison to healthy controls [58]. Another study analyzed baseline CRP levels and prevalence of schizophrenia (with possible hospitalization) in 78,810 Danish males [42]. It found baseline CRP levels in individuals with schizophrenia were 63% higher compared to healthy individuals [42]. A large meta-analysis consisting of 85,000 subjects also found a modest rise in CRP levels in patients with schizophrenia [59]. Along with CRP, some studies have shown higher levels of baseline neutrophils and other innate immunological markers in schizophrenia [25]. In addition, individuals with an elevated baseline CRP (due to any cause such as an autoimmune disease) level had a six to eleven times greater chance of developing schizophrenia late in their adult life [42]. Further evidence from various geographical subgroups linking CRP levels with schizophrenia has strengthened the notion that there is an immune component in active and latent psychosis [60,61,62].

A recent meta-analysis of 21 observational studies involving 7682 subjects demonstrated how CRP is associated with higher suicidality in patients with mental disorders [63]. This could lead to an increase in our understanding of the pathophysiological underpinnings of suicide and improve its prevention [63]. In light of continued identification of environmental and non-genetic risk factors of schizophrenia and psychosis, recognition of patients at “high risk” is becoming increasingly possible. Therefore, serum CRP levels, along with levels of other inflammatory mediators, in high-risk individuals have the potential of becoming a useful tool in predicting the appearance of psychotic symptoms [44]. Furthermore, although scarce, evidence exists for elevated CRP levels being associated with increased mortality in various psychiatric disorders including psychosis [64]. Despite encouraging advances in investigating immune molecules as a marker for predicting psychosis, it is still not conclusively known whether the prominent inflammation shown to coincide with schizophrenia occurs in the premorbid phase too [65]. Currently, evidence found via meta-analyses is still inadequate to use CRP as an accurate predictor to determine the status of which high-risk individuals would “convert” into psychosis [44,65,66].

## 6. CRP and Treatment of Schizophrenia

CRP has been used to assess response to treatment in many psychiatric and non-psychiatric conditions owing to its status as a relatively simple and readily attainable marker of peripheral inflammation [67]. The available clinical data through individual longitudinal studies are largely heterogeneous with varying effects on CRP levels observed after treatment of schizophrenia was initiated via commonly used antipsychotics, such as haloperidol, risperidone, or clozapine [59]. However, some authors review the literature and propose that typical (affecting mainly dopaminergic pathways) and atypical (affecting both dopaminergic and serotonergic pathways) antipsychotics both alleviate the symptoms of acute psychosis partially via reduction of the overall inflammatory state [68]. The classic side effects of long-term conventional antipsychotic use include weight gain and metabolic syndrome and that has been posited to be the reason behind increased inflammation and CRP levels that override their partial anti-inflammatory effect [69]. Fernandes et al. performed multiple meta-analyses on the available clinical data and reported that therapy with solely typical or atypical antipsychotic medication has not been found to reduce or bring about a significant change in CRP levels [59]. This is perhaps owing to the immense importance given to the notion of monoaminergic neurotransmitter disbalance in the causality of schizophrenia [49]. With more clinical data now reported on the inflammatory characteristic of psychosis, it is imperative to review our treatment approach.

The role of CRP in the psychopharmacology of schizophrenia must not be limited just to its position as a marker of recovery or treatment success subsequent to antipsychotic therapy. Early recognition of abnormal CRP levels and identification of high-risk patients may assist and guide therapeutic approaches, including targeted anti-inflammatory medications [67,70]. Therefore, to counter high-grade inflammation denoted by CRP, the use of “add-on” anti-inflammatory pharmacological agents (like non-steroidal anti-inflammatory drugs, or NSAIDs) with traditional antipsychotics in the treatment of schizophrenia becomes necessary. Functionally, the difference among NSAIDs depends on their activity as a COX-1 or COX-2 inhibitor and their subsequent effect on kynurenic acid [68]. Activation of the kynurenine pathway is an important inflammatory sequela that is denoted by a high CRP level peripherally. Models on COX-isoforms in mouse models have revealed that COX-1 inhibitors increase the kynurenic acid levels in the brain while COX-2 inhibitors reduce them [71]. Due to the direct association of increased kynurenic acid in CNS with psychotic symptoms, pharmacological agents are utilized to achieve a reduced kynurenic acid level. Selective COX-2 inhibitors like parecoxib, meloxicam, among others have been shown to decrease brain kynurenic acid levels and possibly treat psychosis [71]. Another drug, celecoxib (a COX-2 inhibitor), has also been investigated and trials reported a significantly improved outcome and better functioning cognitive status in groups that were treated with celecoxib and risperidone (antipsychotic) compared to placebo groups [72]. However, other anti-inflammatory agents have been highlighted in improving schizophrenic symptoms. Clinical trials have suggested agents like acetylsalicylic acid (aspirin) and N-acetylcysteine (NAC) have the potential to reduce some of the debilitating symptoms of schizophrenia [73,74].

Furthermore, a high CRP level in an individual may denote their resistance to antipsychotic treatment [67]. An increasing body of evidence now posits treatment-resistant schizophrenia (TRS) has a distinct pathogenesis compared to treatment-responsive schizophrenia [75,76]. This can be possibly due to the conventional treatment methods attempting to restore dopaminergic balance in the CNS while TRS is not shown to have a prominent dopamine-related pathology [75]. On the other hand, chronic inflammation has been implicated in TRS. A French population study also found an association between TRS, and high levels of peripheral inflammation measured via increased CRP levels [77]. Measuring CRP levels, therefore, may allow clinicians to identify patients at high risk for developing treatment resistance to antipsychotics and in turn identify a cohort that will be a suitable candidate for receiving anti-inflammatory medication [67]. Naturally, this further paves way for anti-inflammatory medication being particularly useful in alleviating negative symptoms in individuals with TRS [77].

## 7. Conclusions and Ways Forward

As a commonly tested biomarker, CRP can have a groundbreaking impact on the diagnosis, management, and prevention of schizophrenia. There is a need to reevaluate and move beyond, our view of CRP as only a conventional biomarker for inflammation. CRP represents a widespread, inexpensive, and easily executable biomarker all over the world, which has long been a fairly non-specific peripheral inflammation assessment tool [78]. Most healthcare settings around the world already perform CRP tests on a daily basis to ascertain multiple clinical conditions [25] and therefore incorporating CRP as a routinely performed test in psychiatric conditions will not add a substantial cost of obtaining new equipment or specialized training of staff. Estimates vary according to region but CRP testing is fairly cost-effective and yields results fairly quickly from a simple serum sample [79]. In fact, many authorities have promoted the use of CRP to test undiagnosed conditions before other tests like erythrocyte sedimentation rate (ESR) [80]. The use of a simple and commonly used biomarker like CRP in assessing schizophrenia has the potential to make an enormous difference in the attitude towards psychotic disorders in resource-strained settings like in healthcare systems of LMICs. Nevertheless, newer inflammatory biomarkers like procalcitonin (PCT) are now also being utilized in place of CRP and ESR. However, the available data for use of PCT in the identification or screening of psychosis is extremely small [81,82] and further empirical data is necessary to compare the efficacy of PCT against other established biomarkers like CRP.

Further studies on CRP will invariably allow for alteration in our therapeutic approach to schizophrenia by validating the addition of an anti-inflammatory agent in the regimen. It is similarly vital to further investigate CRP’s role alone, or in conjunction with other biomarkers, in predicting the conversion of high-risk individuals into those with schizophrenia. It is imperative that more studies are conducted on larger population cohorts to assess more than just the difference of CRP levels between individuals with schizophrenia and healthy controls. Instead, longitudinal studies to investigate altercations of CRP levels over time, especially before and after initiation of antipsychotic and anti-inflammatory treatment, need to be conducted and the efficacy of add-on medications may be checked as well. Within diagnosed schizophrenia patients, CRP levels should be studied in association with the severity of symptoms, relapses, and other comorbidities. This article puts forward an urgent need to collect empirical data and review our understanding of etiology, diagnosis, monitoring, and treatment of schizophrenia.
